# Activated phosphoinositde 3-kinase (PI3Kδ) syndrome: an Italian point of view on diagnosis and new advances in treatment

**DOI:** 10.1186/s13052-024-01662-5

**Published:** 2024-05-20

**Authors:** Vassilios Lougaris, Federico Le Piane, Caterina Cancrini, Francesca Conti, Alberto Tommasini, Raffaele Badolato, Antonino Trizzino, Marco Zecca, Antonio De Rosa, Federica Barzaghi, Claudio Pignata

**Affiliations:** 1 Pediatrics Clinic, Department of Clinical and Experimental Sciences, University of Brescia, Azienda Socio Sanitaria Territoriale Spedali Civili di Brescia, Brescia, Italy; 2 Scientific Department Planning Congressi Srl, Bologna, Italy; 3 Department of System Medicine, Pediatric Chair, University of Tor Vergata, Rome, Italy; 4 Research and Clinical Unit of Primary Immunodeficiencies, IRCCS Bambin Gesù Children Hospital, Rome, Italy; 5 Pediatric Unit, IRCCS Azienda Ospedaliero-Universitaria Di Bologna, Bologna, Italy; 6 Department of Medical, Surgical and Health Sciences, University of Trieste, Trieste, 34137 Italy; 7 Department of Pediatrics, Institute for Maternal and Child Health, IRCCS Burlo Garofolo, Trieste, 34137 Italy; 8 Department of Pediatrics, Università di Brescia, Istituto di Medicina Molecolare Angelo Nocivelli”, ASST Spedali Civili, Brescia, Italy; 9 Department of Pediatric Hematology and Oncology, ARNAS Ospedali Civico Di Cristina Benfratelli Hospital, Palermo, Italy; 10 Paediatric Haematology and Oncology, Fondazione IRCCS Policlinico San Matteo, Pavia, Italy; 11 Department of Translational Medical Sciences, Università degli Studi di Napoli “Federico II”, Naples, 80125 Italy; 12 San Raffaele Telethon Institute for Gene Therapy (Sr-Tiget), Istituto di Ricovero e Cura a Carattere Scientifico (IRCCS) Ospedale San Raffaele, Milan, Italy

**Keywords:** APDS, PI3Kδ, IEI, Leniolisib, Sirolimus, NGS, ESID, EBV, Lymphoma

## Abstract

Activated phosphoinositide 3-kinase (PI3Kδ) Syndrome (APDS) is an inborn error of immunity (IEI) with a variable clinical presentation, characterized by infection susceptibility and immune dysregulation that may overlaps with other Primary Immune Regulatory Disorders (PIRDs). The rarity of the disease, its recent discovery, and the multiform /multifaced clinical presentation make it difficult to establish a correct diagnosis, especially at an early stage. As a result, the true prevalence of the pathology remains unknown. There is no treatment protocol for APDS, and drug therapy is primarily focused on treating symptoms. The most common therapies include immunoglobulin replacement therapy, antimicrobial prophylaxis, and immunosuppressive drugs. Hematopoietic stem cell transplantation (HSCT) has been used in some cases, but the risk-benefit balance remains unclear. With the upcoming introduction of specific medications, such as selective inhibitors for PI3Kδ, clinicians are shifting their attention towards target therapy.

This review provides a comprehensive overview of APDS with a focus on diagnostic and treatments procedures available. This review may be useful in implementing strategies for a more efficient patients’ management and therapeutic interventions.

Main Text.

## Introduction

Inborn Errors of Immunity (IEI), formerly referred to as Primary Immunodeficiencies (PID), are a group of diseases that are characterized by increased susceptibility to infections and sometimes immune dysregulation. To date, more than 500 genes whose alterations are responsible for the occurrence of primary immunodeficiencies have been identified [1, 2].

Among them, APDS is a rare IEI first described in 2013, which in most cases appears de novo in children with healthy parents [3, 4].

The syndrome is caused by heterozygous mutations on PIK3CD (APDS1) and PIK3R1 (APDS2) genes, which code respectively for the catalytic (gain-of-function) and regulatory (loss-of-function) subunits of PI3Kδ that determine a dysregulation of PI3K pathway. This dysregulation leads to immune system abnormalities, including lymphoproliferation, recurrent infections, and autoimmunity. The PI3K family of lipid kinases plays a relevant role in eukaryotic cells: they convert by phosphorylation phosphatidylinositol 4,5-bisphosphate (PIP2) to phosphatidylinositol 3,4,5-triphosphate (PIP3), a key intracellular mediator that triggers the activation of the AKT/mTOR/S6 pathways, which is critical in controlling cell proliferation, differentiation, and growth, particularly in lymphocytes. Dysregulation of the PI3Ks family leads to the onset of various diseases, such as cancers, neurological diseases, and IEI [1, 2, 4].

Literature data show a mean onset age of APDS at 1.6 years [2]. The syndrome is characterized by symptoms that include recurrent respiratory tract infections, bronchiectasis, and benign or malignant lymphoproliferation. Interestingly, 30 out of APDS patients (18%) had asthma as concomitant diagnosis, and this symptom together with respiratory infections, recurrent otitis and lymphoproliferations could characterize the onset of the disease [5]. Typically, during adolescence, autoimmune manifestations including gastrointestinal diseases may also arise [2, 6, 7]. However, due to the scarce knowledge among clinicians of the syndrome, affected patients may be diagnosed late in life, although symptoms may arise early in childhood. Furthermore, the clinical and immunological phenotype may be quite variable. The relevant literature reports on cases of oligosymptomatic or completely asymptomatic patients [2, 6, 8]. The immunological pattern of these patients is marked by an abnormal production of antibodies (high IgM and or IGA, low IgG and IgA), which *leads to Hyper-IgM-like syndrome* [8, 9], with increased susceptibility to recurrent and chronic respiratory infections as well as viral infections such as VZV, CMV, or EBV, that are often prodromal to disease exacerbation [7, 10].

Noteworthy, lymphoproliferation is a hallmark of APDS, manifesting as malignant or non-malignant disease and splenomegaly, often occurring early in life [2]. Indeed around 75% of patients had manifested these symptoms by 10 years [10, 11].

The most severe complication of APDS is lymphoma, predominantly the B-cell type, which occurs in 25% of cases mostly in adult young age. It represents the most frequent cause of death in APDS patients today [11].

In the APDS2 phenotype other signs are more common, as variable growth and mental retardation, microcephaly, facial dysmorphism [1, 2, 12] and hypothyroidism. These symptoms are common to other genetic disorders, which makes the identification of APDS even more challenging for the clinicians.

Therefore, diagnosis is often complex and, sometimes, it comes only after years of distress for the patient. Today, the advanced techniques and Next Generation Sequencing (NGS), is scaling the capability and speed of diagnoses [2, 13].

The heterogeneity of symptoms of patients with APDS also poses a challenge on what is the best treatment approach. Patients are often treated with supportive treatment aimed at symptoms resolution [14]: immunoglobulin replacement therapy and antibiotic prophylaxis to reduce infections recurrence and immunosuppressants (rituximab and/or corticosteroids, m-Tor inhibitors) in case of immune dysregulation. Increasing knowledge on the disease and its pathogenesis is shifting the focus from conventional immunosuppressor drugs to more specific therapy, based on mTOR inhibitors (i.e., rapamycin) and when available, on small molecules, currently under investigation, such as selective inhibitors for PI3Kδ [1, 6, 12].

## Methods

In this literature review we conducted a narrative analysis of a specific theme: Activated phosphoinositide 3-kinase (PI3Kδ) Syndrome (APDS). Our search was conducted between April and December 2023. We used PubMed but also Medline and Science Direct. We used 9 specific key words (APDS, PI3Kδ, IEI, Leniolisib, Sirolimus, NGS, ESID, EBV, Lymphoma) for our search. There are not a lot of literature about Activated phosphoinositide 3-kinase (PI3Kδ) Syndrome (APDS) so we not refuse any paper about diagnostic and treatment. We included 50 scientific articles and 1 AIFA attachment.

## Epidemiology

As discussed above, APDS is a rare primary immunodeficiency first described in 2013, and its incidence is currently unknown. Indeed, to date, data on APDS do not allow determining the real incidence. Its recent description and the heterogeneous phenotype make it difficult to identify patients for APDS genetic testing. To avoid the progression of the disease and the development of related comorbidities, early diagnosis Is crucial.

According to data published in the European Society for Immunodeficiencies (ESID) registry from 2017 to 2023 the number of APDS patients increased from 77 to 170, paralleling the increased awareness [5, 6].

The variation among the data over the past few years highlights that APDS is an underdiagnosed disease with a higher incidence compared to other inborn errors of immunity (IEI) [4, 6]. The latest updates from the ESID registry also show how the analysis of 170 patients with APDS outlines high penetrance and early onset of APDS compared with other forms of Common Variable Immunodeficiency (CVlD). The vast clinical heterogeneity, even among individuals with the same APDS genetic variant, illustrates how poorly genotype predicts phenotype and disease course, thus suggesting that epigenetic alteration may be implicated in the pathogenesis also in this syndrome [15]. The high clinical overlap between APDS and other CID/CVID such as CTLA4 deficiency suggests a considerable convergence of the pathophysiological pathways involved. The organ systems preferentially affected indicate a specific pathophysiology: bronchiectasis is typical of APDS1, while interstitial lung disease and enteropathy are more common in STAT3 GOF and CTLA4 deficiency. Endocrinopathies are more prevalent in GOF STAT3, but growth disorders are also common in both groups, particularly in APDS2. Early clinical presentation is a risk factor for severe disease in APDS [5, 11].

An Italian study by Tessarin et al. reports on 8 patients, all living in Italy, with APDS1. Data from this cohort points out that the clinical and immunological features of APDS1 vary widely, ranging from the lack of symptoms to severe, life-threatening forms. HSCT may be a life-saving option for a very limited number of cases, while therapies targeting the PI3K pathway will most likely become a valuable tool for improving patients’ clinical management and quality of life [14, 16].

Recently, Vanselow, et al. estimates in Germany that APDS is a very rare condition that affects 1–2 subjects per million, thus confirming that it is insidious and difficult to diagnose [2].

Indeed, about 200 patients are registered between Europe and the Middle East, but the exact number is actually unknown because many are either unregistered or undiagnosed [2].

In a real-world study involving 256 patients with APDS, Bonnen and Hanson reported that the major cause of death in these patients was lymphoma. This finding is confirmed by different studies published by Kracker and Durandy in Blood in 2020. The authors argue that lymphoproliferation and B-cell lymphoma are hallmarks of APDS, frequently responsible for patient death [14, 17].

Referring to data presented at the European Society for Immunodeficiencies (ESID) congress, 23 subjects with APDS were identified in Italy. The pathogenic mutations on PI3KCD or PIK3R1 were identified in 19 of these patients, while 4 patients carry a Variant of Uncertain Significance (VUS) and are classified as APDS-Like (APDS-L). The patient cohort includes 9 familial cases attributable to three families.

Speed of diagnosis is therefore, as mentioned above, crucial in order to implement international disease registries.

### Key points

- APDS is a rare primary immunodeficiencies which real incidence is not known.
- According data reported in ESID registry the number of patients increased from 77 to 170 between 2017 and 2023.
- In Italy according the last data there are 23 subjects with APDS.
- Today APDS diagnosis represents a challenge for the medical community.

## Clinical manifestations

APDS is characterized by an extremely heterogeneous phenotype. However, some symptoms are prevalent, and their characterization and identification are particularly relevant to making a diagnosis.

Recurrent, chronic, and or severe infections are the most frequent clinical symptoms, that often result in long hospitalizations. In most cases, these infections affect the sino-pulmonary tract (> 90% of patients), causing, in particular, pneumonia (43%), sinusitis (29%), and otitis (26%). Asthma is reported frequently at the diagnosis, associated to respiratory infections [5]. In addition, bronchiectasis arises in almost all patients [7, 18, 19].

The literature also describes other forms of infections, such as meningitis, osteomyelitis, and dental abscess. The PI3K pathway (altered in APDS disease) plays a crucial role in anti-Herpesvirus defense [7]. Consequently, chronic or persistent Epstein-Barr virus (EBV) and Cytomegalovirus (CMV) infections are quite frequent in APDS patients.

Benign lymphoproliferation is the second most frequent clinical problem (> 65%). It manifests with lymphadenopathy, splenomegaly, and hepatomegaly. Other characteristic features include tonsil, adenoid, and parotid hypertrophy [7]. Adeno-tonsillectomy or repeated admissions in ENT service could be referred in the medical history [9].

33% of patients suffer from autoimmune diseases, of which cytopenia accounts for the majority of cases. Thyroiditis, glomerulonephritis, insulin-dependent diabetes, pancreatic insufficiency, autoimmune hepatitis, arthritis, and pericarditis in patients with APDS have also been reported in the literature, albeit less frequently [7].

Cases of central nervous system disorders, such as developmental delay in cognitive functions and autism spectrum disorders, are described more frequently in patients with APDS2 (26.2%) than in those with APDS1 (9.5%) [7].

In addition to the clinical features, certain immune system abnormalities are also associated with the disease.

### Key points

- APDS is characterized by an extremely heterogeneous phenotype.
- EBV and CMV chronic infections are an important issue in APDS patients.
- Cases of central nervous system disorders are described more frequently in APDS2.
- The frequency of major clinical manifestations is reported in Table 1.

**Table 1:** illustrates the frequency of major clinical manifestations

Clinical manifestations		
Infections		V V V
Benign lymphoproliferation		V V V
Autoimmunity		V V
Malignancy		V V
Short stature (APDS 2)		V (VV)
Neurodevelopmental delay (APDS 2)		V (VV)

• VVV: more than 70% of cases.

• VV: Between 50% and 70% of cases.

• V: less than 50% of cases.

## Immunological features

A study of 53 patients showed that the majority of patients (79%) had increased IgM levels and reduced IgG concentration (43%). It was further observed that in 58% of patients with normal IgG levels there is still a deficiency in certain subclasses, mainly IgG_2_ and IgG_4_. Decreased IgA levels, observed in 50% of cases, is also common in these patients [19]. The syndrome is also characterized by low circulating B cells, due mainly to a blockage in the maturation of this cell lineage [8]. Indeed, an increase of transitional B cell is a specific marker of APDS.

Characterization of the immunological phenotype of APDS patients also showed a variation in the levels of T lymphocyte subsets. Specifically, there is overtime a decrease in the production of naive CD4 + cells and a concomitant increase in memory CD8^+^ cells, resulting in an inverted CD4^+^/CD8^+^ ratio. CD8^+^ T cells often show signs of senescence and exhaustion. These alterations impair the ability of these patients to properly respond to viral infections [2, 16].

Patients with APDS respond differently to vaccines. Indeed, T cell-independent immune response to vaccine such as to pneumococcal antigens is altered in contrast to a T cell-dependent immune response (e.g., tetanus vaccination) [9, 16].

### Key points

- The majority of APDS patients had increased IgM levels, reduced IgG concentration and decreased IgA levels.
- According literature APDS is characterized from an alteration of T-Cell compartment.
- The frequency of major Immunological features is reported in Table 2.

**Table 2:** illustrates the frequency of major immunological features

Immunological features		
Increased IgM levels	V V V	
Reduced IgG concentration	V V	
Reduced IgA concentration	V V	
Inverted CD4^+/^CD8^+^ratio	V V V	
Altered response to T cell-independent vaccine	V V V	

Legends:

• VVV: more than 70% of cases

• VV: Between 50% and 70% of cases

• V: less than 50% of cases

## Diagnosis

To diagnose APDS, the presence of the symptoms and/or immunologic features described above is necessary, but definitive confirmation is obtained only by genetic testing [19].

An overview of the frequency of major clinical manifestations and immunological features is provided in Tables 1 and 2.

One option for genetic analysis is to use NGS technology to identify the mutation that results in the gain of function of PI3Kδ. Another option for genetic testing is Whole Genome Sequencing (WGS), which saves time and money compared to analyzing a single exon (the area that synthesizes the deficient or defective protein) with the Whole Exome Sequencing (WES) technique [20].

Either way, as more genetic tests are performed, there is a risk of identifying Variants of Uncertain Significance (VUS) [2]. In this case, confirmation by a functional assay is required.

Today, several techniques are available in the literature to assess PI3Kδ functionality, including flow cytometry evaluation of intracellular phosphorylation of AKT and S6 in vitro on PBMC, T, B cells and cell lines [13, 21]. These functional tests, as well as the analysis of protein levels by means of Western Blot, could also be used during the follow-up of patients to assess the efficacy of the therapy [22, 23]. However, these procedures are still not standardized and are performed mainly for research purposes.

Based on reported data, recurrent infections begin to occur by the first year, while lymphoproliferation not before the third year of age. Autoimmune symptomatology occurs later in childhood, while the malignant proliferation may manifest at any age, although it most commonly occurs in late childhood/early adulthood (18 years of age on average) [19].

Despite these data, diagnosis almost never comes at the onset of the first symptoms, and is often delayed. This happens predominantly for a delay (on average 10 years and 6 months) of referral to immunological consultation, as reported by Bloomfield et al. [8].

Indeed, it is worthwhile to raise awareness among pediatricians and the other specialists such as gastroenterologists, pneumologists, oncologists, rheumatologists, who most often encounter these patients [2], so that they can recognize the warning signs of APDS and therefore seek consultation with an immunologist.

To this end, the use of national and international registries, such as the ESID registry, is essential to keep the medical community up-to-date on new phenotypic and/or laboratory features that may characterize immunodeficiencies [1, 2].

### Key points

- The presence of condition reported in Table 2 is necessary but not sufficient to diagnose APDS. Definitive confirmation is obtained by genetic testing like: NGS, WGS and WES.
- When there’s VUS risk it’s necessary a functional assay.
- APDS is characterized from a late diagnosis. Probably the main reason it’s the delay of referral to immunological consultation.

## Lymphoproliferation and lymphoma

Non-neoplastic lymphoproliferation was observed in more than 75% of patients with APDS, with onset in pediatric age or young adulthood [24–26]. Mostly, lymphadenopathies, and splenomegaly, tonsil and adenoids hypertrophy.

Benign lymphoproliferation, a common symptom in cases of APDS, can be difficult to distinguish from malignant disease. A careful histological and cytometric evaluation of lymph nodes, bone marrow biopsy, and cerebrospinal fluid is recommended to understand whether it is lymphoid hyperplasia or lymphoma, and choose the appropriate therapy [6, 27, 28].

The two major reviews in the literature regarding the clinical features of patients with APDS reported that the incidence of malignant forms of lymphoproliferation was 13% in patients with APDS1 and 28% in patients with APDS2 [14].

Coulter et al. describe in a cohort of APDS1 development of lymphoma in 7% out of 53, either EBV-positive or negative, although EBV status was not always reported. In addition, one patient had primary cutaneous anaplastic large cell lymphoma [26]. However, the risk of malignancies is likely to increase with age (the cumulative risk of developing cancer at the age of 40 years was calculated as 78% for APDS2 [29].

30% of patients with APDS were found positive for EBV infection [27]. However, EBV-negative lymphomas are more common (19%) than EBV-positive lymphomas (6%) [27].

With respect to pathogenesis, uncontrolled proliferation and malignant transformation of B lymphocytes in APDS are due to both intrinsic and extrinsic B and T cell mechanisms. It is possible to speculate that failure to control PI3K activation pathways in B cells may directly increase cell survival, proliferation and, in some cases, results in malignant transformation facilitated by the impairment of immune surveillance due to reduced T and NK cells cytotoxic activity. Indeed, the CD8T cell premature immunosenescence/exhaustion phenotype and the alterations in NK cells differentiation are both involved in the impairment of cytotoxic activity [14, 27]. In addition, it has been demonstrated an oncogenic role of PI3K, which is directly implicated in B cell transformation [24].

ESID registry (Maccari et al.) reported lymphoma in 14% of patients. Of note, 10 of these cases were associated with EBV. Among this group of APDS patients with lymphoma, 4 also suffered from other malignancies: 2 ovarian tumors, 1 papillary renal cell carcinoma, and 1 malignant submandibular gland tumor [6]. As can be deduced from the reported data, several of these patients had combined forms of lymphomas [26] although, the most common is diffuse large B cell lymphoma [23].

Data on the incidence of lymphoma in the two different forms of APDS are not congruent in all populations. If the study by Maccari et al. emphasized a higher prevalence in form 2, while Garabedian et al. reported that lymphoma rates in their patient cohort were similar in patients with APDS1 (12.3%) and APDS2 (14.3%) [27, 30].

Lymphoma remains the most serious complication of APDS, representing the leading cause of death, followed by stem cell transplant-related complications and recurrent infections [6].

### Key points

- Lymphoma represents the first cause of death in APDS patients so it’s crucial to identify the difference between benign lymphoproliferation (a common symptom in cases of APDS) and malignant disease.
- To date we do not know if Lymphoma is prevalent in one of the two different forms of APDS, in literature it’s not congruent in all populations.

## Drug treatment

The literature regarding APDS is still scarce and full of open questions, as this condition was only characterized in 2013. For this very reason, it is still a largely under-diagnosed disease and patients received variable treatment.

As for treatment, there are currently no approved protocols, but only case reports and literature reviews. The following are the main therapeutic options.

Most of patients received antibiotic prophylaxis with trimethoprim/sulfamethoxazole and/or azithromycin. 6% received long-term antiviral prophylaxis with acyclovir/valacyclovir [5, 26]. Around 10% received antifungal therapy.

APDS treatment guidelines drawn up in Japan in 2023 strongly recommend the use of antibiotic prophylaxis for immunocompromised patients with antibody deficiency. Moreover, these guidelines suggest the use of antiviral prophylaxis combined with regular EBV/CMV monitoring [1].

*Immunoglobulin replacement therapy*: 87% of patients diagnosed with APDS1 and 89% of patients with APDS2 (in many cases even before the confirmed genetic diagnosis of APDS but as early as the first diagnosis of CID) received long-term immunoglobulin prophylaxis, either intravenously or subcutaneously. This presumably resulted in a reduction in the number of severe infections [12, 26]. Recent APDS treatment guidelines, published in Japan, strongly recommend the use [1] of immunoglobulins for immunocompromised patients.

*Immunosuppressants*: 34% of patients with APDS1 manifested autoimmune or autoinflammatory diseases. Of these patients, 30% were treated at least once with immunosuppressive drugs. Patients with autoimmune cytopenia were classified as responder to steroid and rituximab therapy. Rituximab has been effective in the treatment of non-neoplastic lymphoproliferations, such as lymphadenopathy, splenomegaly, and hepatomegaly [12]. Japanese guidelines emphasize the usefulness of these drugs as interventional therapy in individuals diagnosed with APDS [1].

*Rapamycin*: The mTOR inhibitor rapamycin is the most widely used immunosuppressant according to data reported in the ESID registry (used in 26 out of 77 patients) [6]. Notably, Maccari et al. in their work state how Rapamycin is mainly indicated in cases of lymphoproliferation, colitis, and/or cytopenia. Indeed, the use of this drug show an effect in reducing completely or partially lymphadenopathy and hepatosplenomegaly [31] while did not show pharmacologically relevant effects in the treatment of cytopenias and gastrointestinal diseases [5, 32].

It should be emphasized that it should be used with caution by conducting regular checkups on patients because of its many side effects associated with immune system suppression such as granulocytopenia and oral stomatitis [33]. In a single reported case rapamycin seemed to have a synergistic effect with theophylline, an old antiasthma medication with documented non-selective inhibitory action on PI3Kδ [34].

Assessment of the risk-benefit balance of long-term rapamycin therapy alone or together with theophylline does not yet offer clear conclusions [31, 35].

*Selective inhibitors of PI3Kδ*: The literature agrees and is optimistic regarding the use of selective inhibitors of the hyperactivated enzyme. This new class of drugs could be the turning point for the treatment of APDS, as it can act in a targeted manner on the pathogenetic mechanism of the disease [11, 36, 37].

Literature data show that target therapy conducted using leniolisib demonstrates excellent efficacy. Rao et al. provide clinical data from the first 6 patients treated with leniolisib. Improved quality of life was observed in all patients: they all confirmed an increase in perceived energy, or at least a decrease in fatigue [34]. At 12 weeks of treatment, there is an average 40% reduction in lymph node and spleen volume [9]. Improvements are also shown for thrombocytopenia, anemia in one case. Studies also highlight improvements in immune system function, with optimized blood leukocyte counts. Notably, in 5 out of agreed to join the extension of the study from 12 weeks to 9 months and, thanks to the benefits of the drug on the immune system, were able to stop taking immunoglobulins. Further, data indicate that leniolisib is well tolerated in both short-term and long-term studies [35]. There were no serious adverse reactions to the drug [9]. As demonstrated by the recently published OLE study [38], long-term use of leniolisib results in a general improvement in the clinical condition of patients without causing serious adverse reactions. The study shows that out of the 27 patients enrolled and receiving immunoglobulin replacement therapy, 37% were able to gradually decrease the dose, while 6 of them completely discontinued the therapy. These 6 patients had normal levels of IgG, IgE and IgA, and subsequent infections resolved within a normal period of time, without an increase in antibiotic use. B cells in these patients were functioning and capable of producing antibodies that cleared infections [38].

Nemiralisib, also a potent PI3Kδ inhibitor, can be administered by inhalation. The study published by Begg et al. indicates that the safety and tolerability profile of nemiralisib is good and that the most frequently reported side effect is cough. However, the in vivo study did not show evidence of clinical efficacy [39].

Based on the data provided by this study, it is not possible to demonstrate that treatment by inhalation with nemiralisib is effective in patients with APDS. To date, the clinical development of nemiralisib for the treatment of APDS has been suspended [39].

Seletalisib is another active ingredient that induces potent selective inhibition of PI3Kδ, and can be taken orally. Studies conducted on this molecule showed how it was able to inhibit PI3K by monitoring the number of circulating p-S6 + CD19 + B cells, which decreased from baseline to week 12, the first end point of the study and in four patients for a longer time [40]. The first clinical trial on seletalisib, conducted by Diaz et al., presents a cohort of 7 patients. Four of them were classified as responders following the improvement in clinical condition; 1 patient remained stable throughout the trial; two patients had to withdraw from the trial, due to the occurrence of adverse drug reactions. The two patients excluded reported, respectively, an increased hepatic enzyme production and moderate liver damage that was pre-existing the start of the trial. Taking seletalisib for a long time has led, in any case, to clinical improvement for the majority of patients involved in the study (4/5). In particular, there is evidence of partial remission of lymphadenopathy (2 patients), improvement in lung function (1 patient), normalization of thrombocytopenia (1 patient), and improvement in gastric disease (1 patient). By virtue of the results achieved, this active molecule can be considered a therapeutic option for patients with APDS, as well as a possible bridge-therapy for hematopoietic stem cell transplantation, as confirmed in the second clinical trial. However, the data obtained so far will need to be supported by new and larger clinical trials [40]. Data reported were not published because the molecule has been withdrawn before the introduction on market and clinical practice.

Although there is one experimental evidence in a mouse model that a few PI3Kδ inhibitors, as idelasilib or duvelisib, may act on the enzyme Activation-induced cytidine deaminase, resulting in an enhancement of genomic instability [41], all the clinical phase 2 and phase 3 studies thus far reported [9, 35, 42] only reported mild side effects in patients treated with leniolisib [43]. Adverse events included aphthous ulcers, transient alopecia, taste disorder and vomiting, none being serious. In addition, Leniolisib is structurally unique relative to other approved PI3Kδ inhibitors. Instead of binding to the specificity pocket, leniolisib uses a tryptophan shelf and stacks with Trp760 in p110δ, whereas the corresponding interaction with Trp780 in p110α is prevented, thus conferring specificity for the δ isoform [42]. Leniolisib appears to be much more specific to the δ isoform than the λ isoform, unlike Idealisib [40, 42, 43].

### Key points

- To date there is not an approved protocol for APDS treatment but only an empiric approach based on symptoms.
- In literature we find some main options used for APDS symptoms:
  i) Antimicrobic prophylaxis: Most of patients received antibiotic prophylaxis with trimethoprim/sulfamethoxazole and/or azithromycin. Someone of them received long-term antiviral prophylaxis with acyclovir/valacyclovir.
  ii) Immunoglobulin Replacement Therapy.
  iii) Immunosuppressant: corticosteroids or Rituximab.
  iv) M-Tor inhibitor: Rapamycin is useful for lymphoproliferation but not show pharmacologically relevant effects in the treatment of cytopenia and gastrointestinal diseases.
- Selective Inhibitors of PI3Kδ are on clinical trial. Leniolisib could be on clinical practice soon, it should be the turning point for the APDS treatment.

## Hematopoietic stem cell transplantation (HSCT)

EBMT guidelines with respect to HSCT in IEI strongly recommend that all patients with IEI undergo transplantation only in highly specialized centers and contribute to the compilation of registries, such as EBMT, ESID, and SCETIDE, in order to monitor patients’ outcome data continuously. The decision to perform HSCT or not should take into account multiple factors, including clinical presentation, genotype, immunophenotype, autoimmune reactions, and social factors such as quality of life and fertility [44].

The inability to reduce or otherwise avoid severe infections despite the intravenous administration of immunoglobulins (in addition to an optional antibiotic prophylaxis), coupled with the risk of recurrent and potentially life-threatening lymphoproliferative episodes that do not respond adequately to immunosuppressants, constitutes the rationale for performing HSCT in patients with APDS [45]. According to guidelines in Japan, the use of HSCT could be considered in cases where there is a massive dysfunction or dysregulation of T lymphocytes, a severe lymphoid hyperplasia, or in cases of malignant lymphoma [1].

Nademi et al. [46] describes 11 patients with APDS undergoing HSCT. The age of these patients ranges from 5 to 23 years. Of these, 9 are alive and have regular post-transplant follow-ups. One died 75 days after the treatment due to progressive multi-organ dysfunction. Another patient died 70 days after transplantation from acute lung failure.

Of interest is the work of Dimitrova et al., which compares the outcome of HSCT between APDS1 and APDS2 showing the potential efficacy of hematopoietic stem cell transplantation in reversing the disease phenotype. In 83% of cases, immunoglobulin treatment at 2 years after HSCT was no longer required [47]. The majority of symptoms of APDS, such as infections, lymphoproliferation, hypogammaglobulinemia, and enteropathy, are significantly improved after HSCT [47].

According to the reported data, there is no significant difference in treatment success whether the patient is affected by form 1 or form 2, in relation to the type of donor (a relative or matched unrelated) or to the intensity of the conditioning regimen. Also, it is important to point out that cumulative data reveal that the incidence of stem cell graft failure increases significantly when mTOR inhibitors are administered 1 to 3 years after transplantation [47].

However, it is necessary to pay close attention to the side effects that may arise. According to Okano et al., adverse reactions occur in 90.9% of cases, while engraftment failures in 36.4%. Based on these data, caution is advised in undertaking this therapeutic strategy [48].

Yang X et al. reports on the case of a 6-year-old patient undergoing HSCT from an haploidentical donor (her brother). At 30 days after surgery, bone marrow biopsy confirmed the success of the procedure, with normalization of hematopoiesis. The patient later showed acute episodes of graft instability that improved with the use of immunosuppressants and did not recur. The patient’s quality of life improved markedly, and there were no further episodes of recurrent infections, diarrhea or lymphoproliferations [49].

In conclusion, it has been demonstrated a good efficacy of HSCT therapy although it should be undertaken in the presence of serious symptoms such as acute episodes of lymphoproliferation (even malignant), severe recurrent infections or severe lung disease. In presence of fully HLA matched donor could be indicated also in less severe cases. The high frequency of adverse reactions and episodes of engraftment failure suggest the need to optimize conditioning and patient preparation procedures for surgery. To date, it still remains to develop guidelines for the selection of patients for whom HSCT is appropriate and to define the correct timing and intensity of the procedure.

### Key points

- The majority of symptoms of APDS, such as infections, lymphoproliferation, hypogammaglobulinemia, and enteropathy could be improved with HSCT.
- Based on literature data caution is advised in undertaking this therapeutic strategy. The High frequency of engraftment failure in APDS patients suggest the need to optimize conditioning for surgery.
- The evidence suggests not using m-Tor inhibitors between 1 and 3 years after HSCT.

## Discussion

The aim of this review is to provide an expert opinion, in an attempt to answer some of the key questions based on the studies reported in the literature regarding the recognition, the diagnosis and treatment of APDS, a complex disorder first described in 2013.

The Authors consider that it is mandatory to first introduce awareness program among pediatricians and general practitioners, who should be able to identify potential patients with IEI with a particular attention paid to IEI with immune dysregulatory features. Once the general practitioner has recognized a possible case of immunodeficiency, they will refer the patient to second-level centers. To do that, it is important to define some identifiable clinical features, for example, the presence of recurrent/severe infections, and early autoimmune, lymphoproliferative manifestations. In the presence of such clinical picture, the first-level center will be responsible for performing a preliminary blood test including immunoglobulin serum levels. At the second/third-level center more extensive immunological investigations (e.g., extended immunophenotyping, next generation sequencing and intracellular protein phosphorylation assay) should be conducted.

It is therefore essential, according to the experts, to establish two levels of awareness.

The first is aimed at raising attention for early recognition of clinical signs resulting from immune system deregulation and is addressed to general practitioners or practitioners from first-level centers. The second, more thorough, concerns the second-level centers that will be able to carry out the completion of the diagnostic process. This should reduce the rate of underdiagnosis. Improvement knowledge on the disease and diffusion of information on how recognize. It should be mentioned that patients with IEI, and in particular APDS, may have clinical features predominantly involving a specific apparatus, and thus they may be intercepted by different medical specialists, as gastroenterologists, pulmonologists, oncologists, hematologists, but also endocrinologists and otolaryngologists.

Regarding functional assays to analyze PI3K activity, experts agree that there is currently no established, standardized single test for APDS and it should be performed in those centers that already have a consolidated technical expertise. Nevertheless, experts unanimously argue that, if the patient’s phenotype and detected mutation are congruent, it is possible to make a definite diagnosis even without functional testing. This latter would be strictly necessary only when there is no congruence between genotype and phenotype or if the mutation detected is of unknown significance.

When interviewed about the possibility of identifying prognostic markers of disease severity, experts agree that it is not currently possible to define laboratory or clinical predictive biomarkers. However, the patient’s age and the clearance from infections, in particular EBV, in the subject’s medical history, seem to be important factors.

Regarding the complications of the disease, there is unanimity in designating lymphoma as the worst-case scenario. Furthermore, experts agree that, looking at the natural history of the disease, there is a high probability that all patients could experience this complication, given the cumulative incidence of 78% reported in the literature [27] for APDS 2.

The Authors also believe that it is necessary to provide a specific follow-up plan for these patients to monitor the evolution of lymphoproliferation, given that patients single PET scans can be of difficult interpretation in patients with APDS. To this end, it is critical to build a multidisciplinary team that involves a hematologist, a pathologist and a radiologist. One option would be to schedule periodic ultrasound scans. All experts agree that examinations involving radiation exposure should be avoided during follow-up. They also believe that histological testing should be repeated in case of non-response to treatment or a relapse during follow-up.

Regarding drug therapy, it is a common view that the use of antibiotic prophylaxis should be individualized and limited when possible. Therefore, the optimal strategy is not to recommend prophylaxis but to proceed according to the clinical and immunological picture. Experts believe that this treatment option is appropriate only in cases of severe T cell compartment immunodeficiency. Moreover, only drugs such as cotrimoxazole and azithromycin should be used for this purpose, the latter for its anti-inflammatory property resulting in improvement of pulmonary disease in other chronic disorders such as cystic fibrosis and other IEI. Experts also agree on excluding antivirals such as acyclovir and ganciclovir from therapy for APDS. In most cases, this disease is marked by susceptibility to the EBV, which is not sensitive to these types of antivirals. Concerning antimicrobial therapy, therefore, the optimal strategy is not to recommend prophylaxis but to proceed with a clinical evaluation of each individual case. Instead, it is recommended to vaccinate patients against encapsulated germs as long as there is a residual antibody response to vaccines.

Albeit PI3Kδ inhibitors are showing efficacy in normalizing the immune system of treated patients, experts questioned on this matter were skeptical about the possibility of suspending immunoglobulin therapy in treated patients. There is consensus that the current clinical data are insufficient to consider Leniolisib capable of restoring normal immune system function to the point where immunoglobulin replacement therapy can be discontinued completely, especially if treatment is started later during pediatric age. However, experts consider the possibility of reducing immunoglobulin administrations. This would allow improving patients’ quality of life and complying with European guidelines that call for limiting the number of administrations whenever possible. Currently, there is a global shortage of immunoglobulins due to approval for new indications for use, increasing off-label use, and uncertainty regarding the duration of treatment [50].

With regard to HSCT, it is complex to make a true risk-benefit assessment. Most patients with APDS admitted for transplantation had already compromised clinical conditions, and this certainly biased the outcome data. Therefore, it is desirable to define more clearly which type of patient should undergo transplantation.

Based on the considerations made by experts and the data available to date on APDS, it is not possible to determine a clear patient’s phenotype for which transplantation is indicated. However, it is possible to develop a selection algorithm based on the genetic characteristics of the patient and the potential donor. If the patient with APDS has an HLA-identical and APDS-negative sibling, then the patient is the ideal candidate for transplantation. However, it is necessary to take into account that the birth rate in Italy is low and that the disease has a dominant nature. Thus, it is estimated that such a favorable condition is met in approximately one in every 15 cases. Transplantation should be strongly considered if there is a 10/10 Matched Unrelated Donor (MUD), a possibility that is estimated at 3 out of 15 patients. For all patients who do not fall into one of these two subgroups, transplantation is conceivable only in the case of non-response to drug therapy.

## Conclusions

This literature review highlighted the clinical and immunologic features of APDS, a rare and recently described condition. The review provided insight into how there are still many open questions, especially regarding the correct diagnostic approach and drug therapy. Contributions from some of Italy’s leading experts in the field offered insight into the current situation of the disease in Italy. To date, there are 23 patients diagnosed with APDS in Italy; however, this number is expected to increase in the future, so raising awareness about this condition is crucial. In this regard, it will be essential to define a process to raise awareness among the medical professionals about IEI in general. The management of patients with APDS involves the administration of symptomatic therapy with antimicrobial prophylaxis, replacement therapy with immunoglobulins and immunosuppressants, and, increasingly, the use of rapamycin as target therapy. The possibility of using selective inhibitors for PI3Kδ in the future, such as leniolisib, will provide a greater opportunity to appropriately treat APDS patients. In the future, new evidence will provide a clearer understanding of the clinical efficacy in the long-term follow-up of this selective inhibitor.

It is sadly known that one of the dogmas related to the difficulty of diagnosis in rare diseases is that “clinicians look for what they know.” This is even more evident in very rare diseases like APDS. Therefore, awareness raising and appropriate training of specialist doctors who may first come across these symptoms is crucial for the diagnosis and early treatment of these patients.

## Data Availability

Not Applicable.

## Declarations

N.V. for their own contribution to writing of this article. Author RB has declined an.

honorarium for his contribution to this manuscript.

## Abbreviations

APDS: Activated Phosphoinositide 3-Kinase Syndrome
IEI: inborn error of immunity
PIRD’s: Primary Immune Regulatory Disorders
PID: Primary Immunodeficiencies
HSCT: Hematopoietic Stem Cell Transplantation
CVID: Common Variable Immunodeficiency
VUS: Variant of Uncertain Significance
ESID: European Society for Immunodeficiencies
NGS: Next Generation Sequencing
WGS: Whole Genome Sequencing
WES: Whole Exome Sequencing
EBV: Epstein-Barr virus
CMV: Cytomegalovirus
GOF: Gain of Function
